# Supplementary material for: Dynalign II: common secondary structure prediction for RNA homologs with domain insertions
Source: Nucleic Acids Res. 2014 Nov 21;42(22):13939–48. doi: 10.1093/nar/gku1172 (PMC4267632; doi:10.1093/nar/gku1172)
Supplement: SUPPLEMENTARY DATA [file supp_gku1172_nar-02021-z-2014-File012.zip › manual/RNA_class/html/globals_func.html]

RNAstructure Classes: Class Members

- Main Page
- Classes
- Files

- File List
- File Members

- All
- Functions
- Variables
- Defines

- errmsg()
  : RNA.cpp- main()
    : Dynalign\_class.cpp
    , RNA\_class.cpp
    , OligoWalk\_class.cpp
    , HybridRNA\_class.cpp
    , DynalignSMP\_class.cpp

---

Generated on Tue Jul 30 10:52:29 2013 for RNAstructure Classes by 
 1.5.7.1
